# USP18 promotes clear cell renal cell carcinoma progression by regulating the ubiquitination and stability of YBX3

**DOI:** 10.1016/j.isci.2026.115808

**Published:** 2026-04-17

**Authors:** Chen Wang, Yihui He, Zhijie You, Siqi Chen, Xin Chen, Xin Chen

**Affiliations:** 1 Department of Pathology, Shengli Clinical Medical College of Fujian Medical University, Fujian Provincial Hospital, Fuzhou University Affiliated Provincial Hospital, Fuzhou, Fujian 350001, China; 2 Department of Pathology, Fujian Provincial Hospital South Branch, Fuzhou University Affiliated Provincial Hospital, Fuzhou, Fujian 350001, China

**Keywords:** biological sciences, cell biology, molecular biology

## Abstract

Y box binding protein 3 (YBX3) is an oncogene in clear cell renal cell carcinoma (ccRCC). This study aimed to investigate ubiquitin-specific proteases (USPs) that regulate YBX3 stability in ccRCC. USP18 was identified as a potent stabilizer of YBX3, and USP18 was depleted to assess its impact on the malignant behavior of ccRCC cells and on YBX3 ubiquitination. Subsequently, YBX3 was overexpressed in USP18-deficient cells to determine whether it could rescue the attenuated malignant phenotypes. A xenograft model and ccRCC organoids were also used to examine the effect of USP18 on ccRCC. USP18 knockdown caused reduced viability, arrested cell cycle, increased apoptosis, attenuated migration and invasion of ccRCC cells. Mechanistically, USP18 reduces ubiquitination and stabilizes YBX3 in ccRCC cells. *In vivo*, USP18 deficiency inhibits xenograft tumor growth by decreasing YBX3 expression and blocking the PI3K/AKT pathway. Our findings underscore the therapeutic potential of targeting the USP18-YBX3 axis in ccRCC treatment.

## Highlights

• USP18 is overexpressed in ccRCC tissues and cells
• USP18 stabilizes YBX3 protein by reducing its ubiquitination in ccRCC cells
• YBX3 overexpression restore the reduced malignancy of USP18-deficient ccRCC cells

## Graphical abstract

**Figure:**
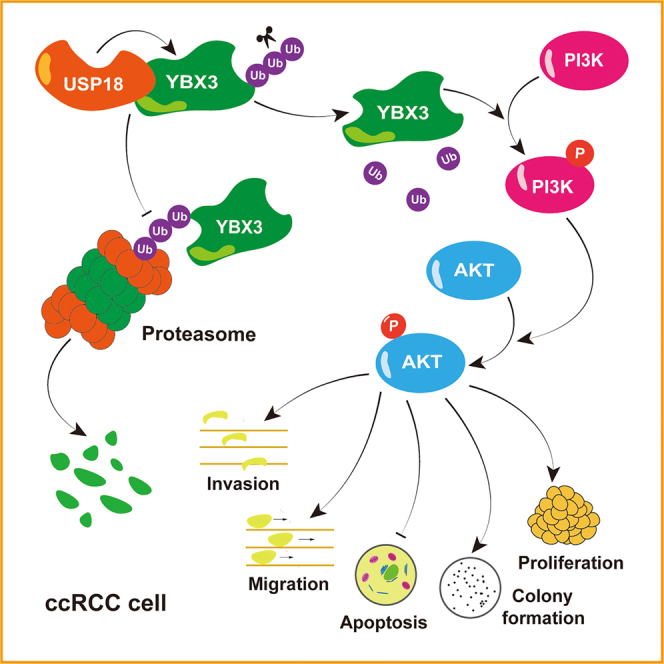

## Introduction

Renal cell carcinoma (RCC) is a highly heterogeneous malignancy, ranking second among all urological cancers. Among RCC subtypes, clear cell RCC (ccRCC) is the most common, accounting for 85%–90% of all renal cancers.^1^^,^^2^ The diagnosis of ccRCC is hindered by a lack of significant clinical manifestations. Surgery remains the primary treatment for ccRCC. However, patients with advanced ccRCC often have a poorer prognosis due to high rates of metastasis and mortality.^3^^,^^4^ While targeted therapies for ccRCC have been implemented, their efficacy remains limited.^5^^,^^6^ Thus, it is imperative to explore the underlying mechanisms of ccRCC progression and identify more effective treatment strategies.

Ubiquitination, a major pathway regulating protein degradation, is accomplished through stepwise enzymatic reactions involving the E1-E2-E3 cascade. This process can be reversed by deubiquitinases (DUBs).^7^^,^^8^ Accumulating evidence has shown that DUBs function as essential regulators of various cancer-related signal pathways, and their dysregulation contributes to tumorigenesis. Therefore, DUBs are considered promising druggable targets in anti-cancer treatment.^9^^,^^10^ The ubiquitin-specific proteases (USPs) form the largest subfamily of DUB,^11^^,^^12^ and the role of multiple USPs in ccRCC has been documented. For instance, USP13 and USP37 facilitate ccRCC tumorigenesis by enhancing the stability of ZHX2 and HIF2α, respectively.^13^^,^^14^ However, the impact of other USPs in ccRCC and the underlying mechanism require further characterization.

The PI3K/AKT pathway is a critical signaling pathway that regulates a diverse array of biological processes, such as proliferation, cell cycle, and apoptosis,^15^ and its role in tumorigenesis is also well characterized.^16^ In ccRCC, genetic alterations in genes associated with this pathway, such as *PIK3CA*, *PTEN*, and *AKT*, have been discovered in a significant portion of patients with ccRCC.^17^ Nevertheless, the mechanism underlying the dysregulation of the PI3K/AKT pathway, particularly at the posttranslational level, remains inadequately elucidated.

Y box binding protein 3 (YBX3) belongs to the YBX family whose critical role in tumorigenesis has been well-documented.^18^^,^^19^^,^^20^ Among these, YBX3 is a key factor regulating protein synthesis and is intricately related to a variety of tumor types. For example, YBX3 promotes the metastasis of nasopharyngeal cancer via activating the PI3K/AKT pathway.^21^ Moreover, the expression level of YBX3 is negatively associated with the overall survival of hepatocellular carcinoma.^22^ In ccRCC, high YBX3 expression correlates with more advanced stages and poor prognosis,^23^ and could serve as an independent prognostic marker of ccRCC.^24^ However, the mechanism controlling YBX3 expression and protein stability in ccRCC is incompletely understood.

Here, we initially screened USPs that may stabilize YBX3 and identified USP18 as a potent stabilizer. Next, the expression of USP18 in the tissues and cell lines of ccRCC was detected. We then depleted USP18 to investigate the impact of USP18 deficiency on the malignant behaviors of ccRCC cell lines. Additionally, we studied the interaction between USP18 and YBX3 in ccRCC cells and assessed the influence of USP18 on the ubiquitination and stability of YBX3. Furthermore, we overexpressed YBX3 in USP18-deficient ccRCC cells and evaluated viability, proliferation, cell cycle, apoptosis, migration, and invasion. Then, we explored the impact of USP18 deficiency on ccRCC progression *in vivo* using a xenograft mouse model. Finally, we also cultivated ccRCC organoids and verified the function of USP18.

## Results

### USP18 is a potent stabilizer of YBX3

**Figure 1:**
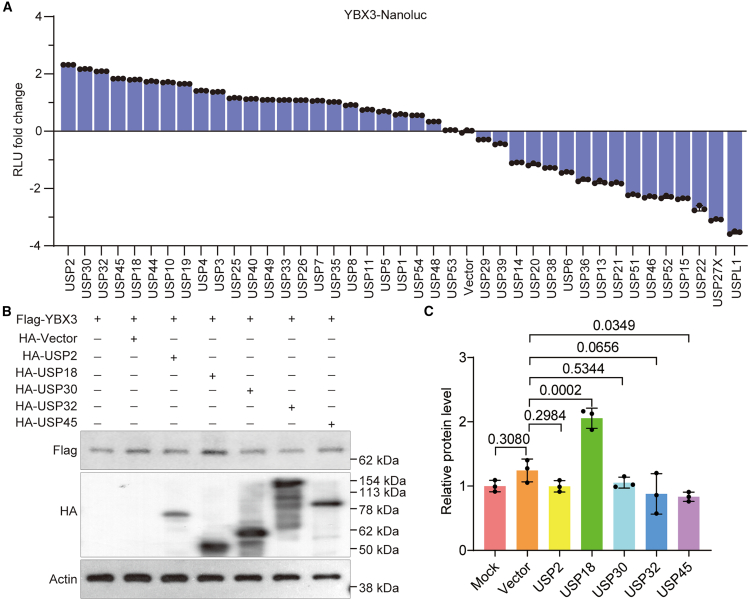
Screening of USPs that may regulate the stability of YBX3 (A) Dual-luciferase reporter assay results show that 40 USPs affected the stability of luciferase-conjugated YBX3. (B) Western blotting data depict the influence of overexpressed USP2, USP18, USP30, USP32, and USP45 on YBX3 protein levels in HEK293T cells. (C) Statistical analysis of the results of (B), band intensities were analyzed using ImageJ. One-way ANOVA followed by Tukey’s multiple comparison test was utilized for significance evaluation. The experiments were repeated three times independently. Error bars in Figures 1A and 1C indicate standard deviation. Data are represented as mean ± SD.

To screen for USPs that may enhance the stability of YBX3, we utilized a luciferase activity-based strategy. By fusing the *YBX3* coding sequence with a luciferase reporter and assessing luciferase activity, we identified several USPs that augment the activity of *YBX3*-Nanoluc, including USP2, USP30, USP32, USP45, and USP18, among others (Figure 1A). However, western blotting revealed that only USP18 significantly augmented the expression of YBX3 in HEK293T (Figures 1B and 1C). These findings indicate that USP18 can enhance the stability of YBX3 and may have implications in ccRCC.

### USP18 is overexpressed in ccRCC

**Figure 2:**
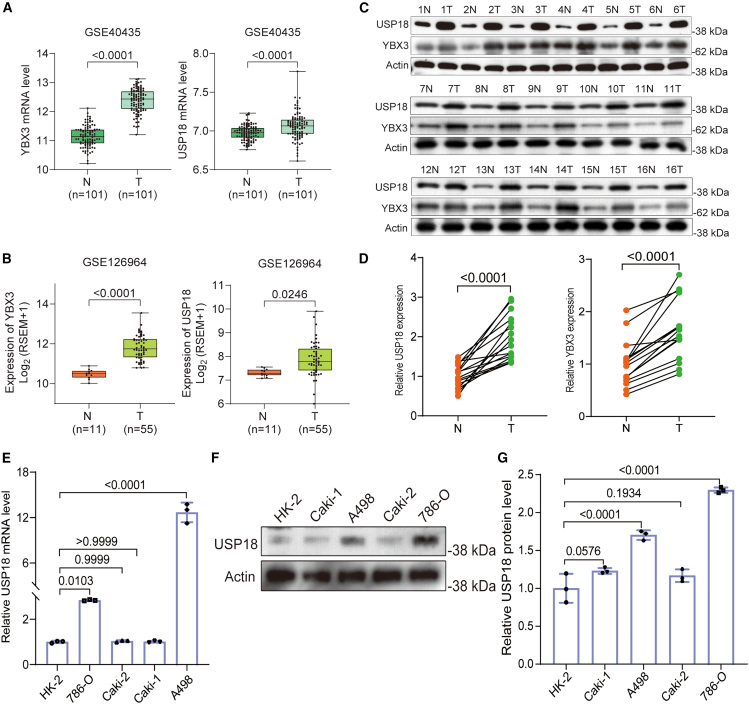
YBX3 and USP18 levels are elevated in ccRCC (A and B) Bioinformatic analysis data reveal higher mRNA levels of *YBX3* and *USP18* in ccRCC compared to adjacent non-cancerous tissues, with statistical analysis conducted by unpaired two-tailed Student’s *t* test. (C) Western blotting results demonstrate elevated protein levels of YBX3 and USP18 in ccRCC (T) compared to adjacent non-cancerous tissues (N), analyzed using paired two-tailed Student’s *t* test. (D) Statistical analysis of the results of (C), band intensities were analyzed using ImageJ. qPCR (E) and Western blotting (F, G) data indicate the overexpression of *USP18* in 786-O and A498, but not in Caki-1 and Caki-2, relative to HK-2 cells. One-way ANOVA followed by Tukey’s multiple comparison test was used for significance calculation. The experiments in Figures 2E and 2F were repeated three times independently. Error bars in Figures 2E and 2G indicate standard deviation. Data are represented as mean ± SD.

To probe the influences of USP18 in ccRCC, we initially compared its expression in ccRCC tumors with adjacent non-cancerous tissues using two ccRCC transcriptomic datasets. Bioinformatic analysis revealed a significant upregulation of both *YBX3* and *USP18* in ccRCC compared to non-cancerous tissues (Figures 2A and 2B). To validate this observation, we collected ccRCC samples and assessed USP18 and YBX3 expression by western blotting. The results demonstrated higher levels of both USP18 and YBX3 in ccRCC compared to non-cancerous renal tissues (Figures 2C and 2D). Consistently, both *USP18* mRNA and protein expressions were markedly elevated in A498 and 786-O, compared to HK-2 cells, as demonstrated by qPCR and western blotting (Figures 2E–2G). Given the observed correlation between increased USP18 expression and elevated YBX3 levels, coupled with the carcinogenic effects of YBX3 in ccRCC, these findings suggest a potential role for USP18 in promoting ccRCC progression.

### Knockdown of USP18 impairs the malignant behaviors of ccRCC cells

To confirm the potential oncogenic role of USP18 in ccRCC, we initiated knockdown experiments targeting USP18 in ccRCC cells. Initially, we designed three shRNAs targeting *USP18* and assessed their efficiency in HEK293T cells. qPCR results demonstrated that all three shUSP18 constructs efficiently reduced *USP18* expression (Figure S1A). Among these, shUSP18-3 exhibited the most effective knockdown efficiency and was selected for subsequent experiments. Consistently, efficient depletion of *USP18* was also observed in ccRCC cells (Figure S1B). Notably, reduced USP18 expression minimally impacted *YBX3* mRNA expression in these ccRCC cells, suggesting that USP18 regulates YBX3 expression at the posttranslational level.

**Figure 3:**
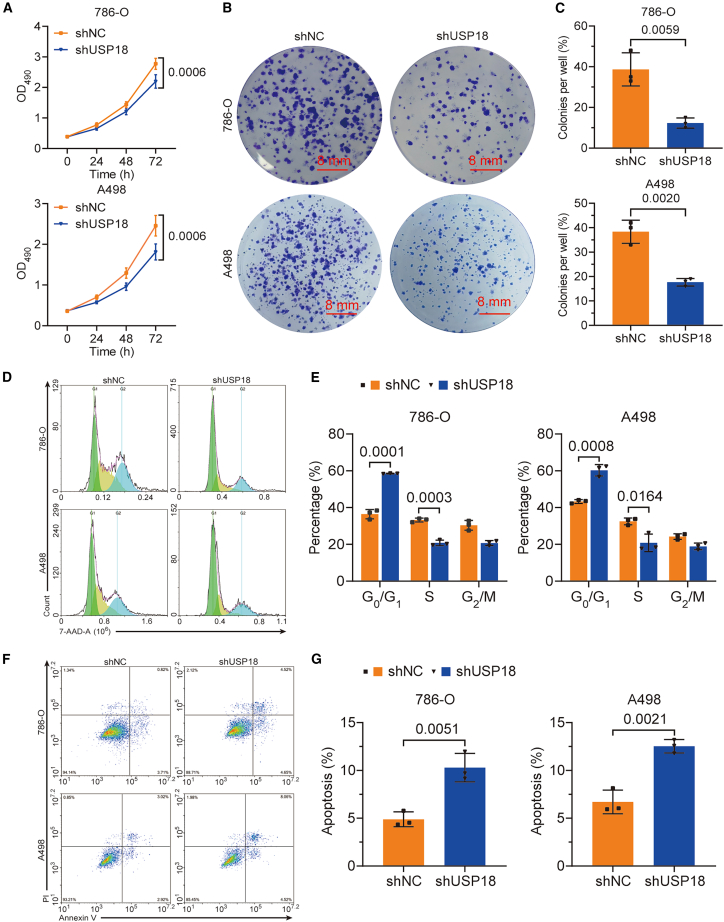
Knockdown of USP18 inhibits viability, mitigates colony formation capacity, and reduces cell cycle progression, but promotes apoptosis in ccRCC cells (A) MTT data reveal that knockdown of USP18 reduces the viability of ccRCC cells. (B and C) Results from the colony formation demonstrate that USP18 knockdown decreases the proliferation of ccRCC cells (scale bars, 8 mm). (D and E) 7-AAD assay uncovers an increase in cells arrested at the G0/G1 stage and a reduction in cells entering the S stage upon USP18 deficiency. (F and G) Knockdown of USP18 promotes apoptosis in 786-O and A498, as evidenced by Annexin V/PI staining results. Unpaired two-tailed Student’s *t*-tests were employed to determine the significance. The experiments were repeated three times independently. Error bars in Figures 3A, 3C, 3E, and 3G indicate standard deviation. Data are represented as mean ± SD.

**Figure 4:**
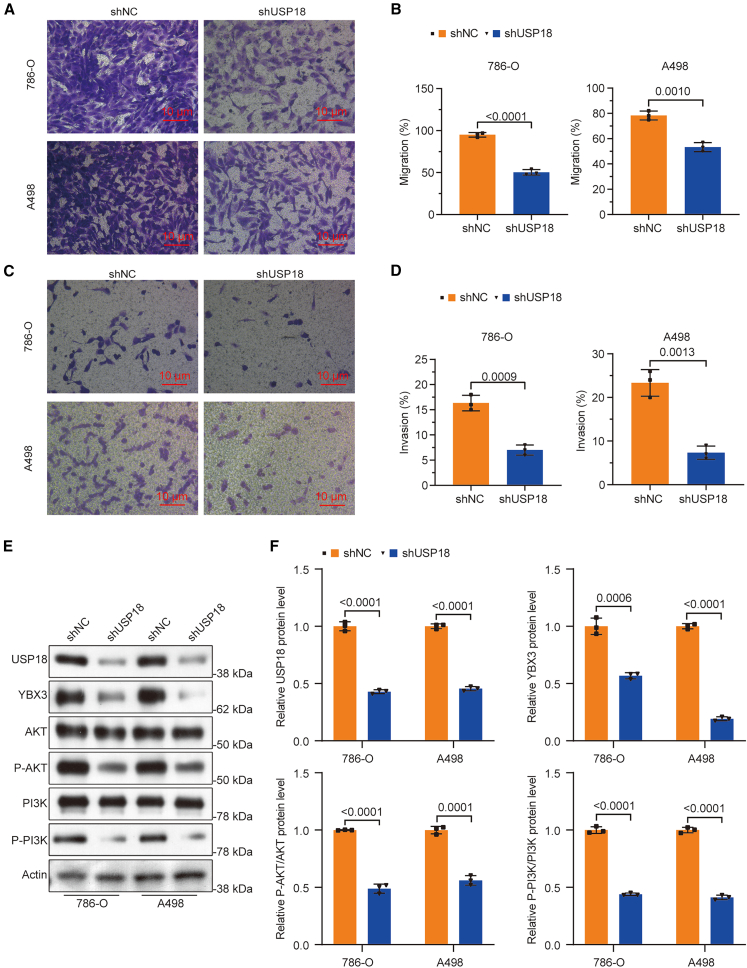
USP18 depletion attenuates the migration and invasion of ccRCC cells and decreases the expression of P-PI3K and P-AKT (A and B) Results from the Transwell assay demonstrate that knockdown of USP18 impairs the migration of ccRCC cells (scale bars, 10 μm). (C and D) Transwell assay data reveal that USP18 deficiency decreases the invasion of ccRCC cells (scale bars, 10 μm). (E and F) Western blotting reveals reduced levels of YBX3, P-AKT, as well as P-PI3K in ccRCC cells upon USP18 depletion. Unpaired two-tailed Student’s *t*-tests were used for significance calculation. The experiments were repeated three times independently. Error bars in Figures 4B, 4D, and 4F indicate standard deviation. Data are represented as mean ± SD.

Next, we evaluated the impact of USP18 deficiency on the malignant behaviors of ccRCC cells. MTT results showed that USP18 knockdown lessened the viability of ccRCC cells (Figure 3A). In line with this finding, colony formation assay outcomes uncovered that USP18 silencing attenuated the proliferation of ccRCC cells (Figures 3B and 3C). The depletion of USP18 also led to more cells arrested in the G_0_/G_1_ stage and fewer cells entering the S stage in ccRCC cells, as indicated by the 7-AAD staining assay (Figures 3D and 3E). Furthermore, Annexin V/PI staining revealed that USP18-deficient 786-O and A498 cells are more apoptotic than their control counterparts (Figures 3F and 3G). Additionally, the migration and invasion of ccRCC cells were also mitigated upon USP18 knockdown (Figures 4A–4D).

Considering the impacts of USP18 in activating the PI3K/AKT pathway in other contexts,^25^^,^^26^ we performed a bioinformatic analysis on genes whose expression is associated with *USP18* levels in the GSE126964 dataset. The results revealed that genes differentially expressed between the USP18-high and USP18-low groups (classified based on the average *USP18* mRNA level) demonstrated a significant positive correlation with the PI3K pathway (Figure S2). Next, we examined the levels of P-PI3K and P-AKT in ccRCC cells upon USP18 depletion. Western blotting demonstrated a significant reduction in P-PI3K as well as P-AKT levels in 786-O and A498 cells upon USP18 knockdown (Figures 4E and 4F). Remarkably, decreased USP18 protein levels correlated with the lower expression of YBX3 protein in these cells, further supporting the role of USP18 as a YBX3 stabilizer in ccRCC. Together, these findings indicate that USP18 functions as an oncogene in ccRCC.

### USP18 stabilizes YBX3 protein by reducing its ubiquitination in ccRCC cells

**Figure 5:**
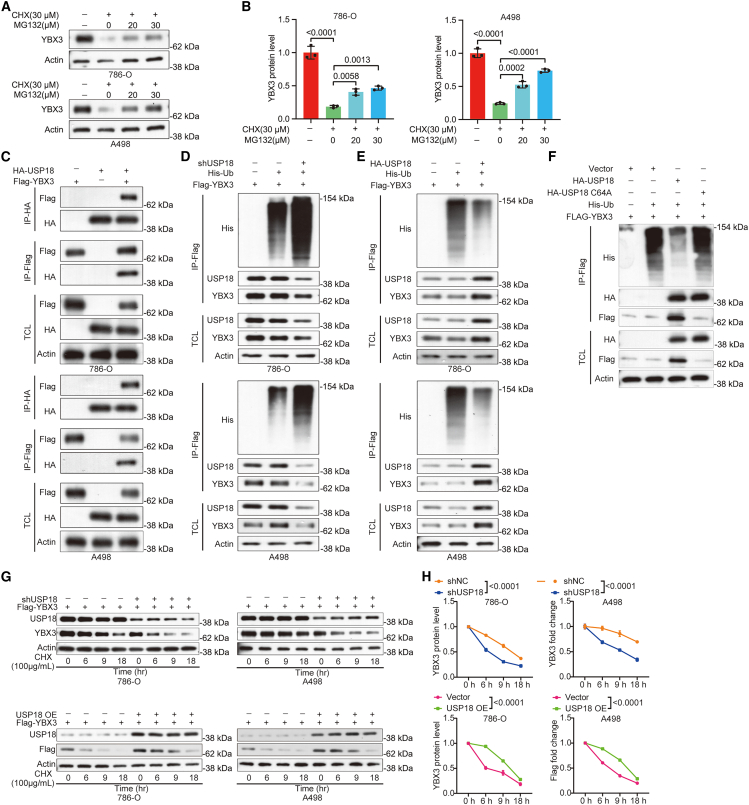
USP18 interacts with YBX3 and negatively controls its ubiquitination in ccRCC cells (A and B) Western blotting data reveal that compared with 786-O and A498 cells not treated with CHX, CHX treatment significantly reduces the protein levels of YBX3. However, the addition of MG132 alleviates the degradation of YBX3 in a dose-dependent manner. Cells were treated with or without CHX (30 μM) and MG132 (0, 20, 30 μM) for 6 h. One-way ANOVA followed by Tukey’s multiple comparison test was used for *p*-value calculation. (C) Co-IP results demonstrate the interaction between USP18 and YBX3 in 786-O and A498 cells. (D and E) Depletion (D) or overexpression (E) of USP18, respectively, enhances or mitigates YBX3 ubiquitination in 786-O and A498 cells, as indicated by IP results. (F) IP data show that *USP18* C64A could not deubiquitinate YBX3 in 786-O cells. (G and H) Western blotting data reveal that knockdown of USP18 accelerates YBX3 degradation, while excessive USP18 increases the stability of YBX3. shUSP18 or *USP18* OE and Flag-*YBX3* plasmids were transfected into cells, cells were then incubated with 100 μg/mL CHX for 0, 6, 9, and 18 h, respectively. TCL, total cell lysate. Two-way ANOVA followed by Sidak’s post hoc test was used. The experiments were repeated three times independently. Error bars in Figures 5B and 5G indicate standard deviation. Data are represented as mean ± SD.

To elucidate the mechanism by which USP18 promotes ccRCC malignancy, we investigated its interaction with *YBX3*, an oncogene in ccRCC, as previously mentioned. Initially, we treated 786-O and A498 with a protein synthesis inhibitor, CHX, to halt further YBX3 protein synthesis, while concurrently administering different dosages of the proteasome inhibitor, MG132, to these cells. Western blotting revealed a dose-dependent increase in YBX3 protein levels upon MG132 treatment (Figures 5A and 5B), confirming the role of the ubiquitin-proteasome system in controlling YBX3 degradation in 786-O and A498.

Subsequently, we conducted Co-IP assays to investigate the reciprocal interaction between USP18 and YBX3 in ccRCC cells. The results demonstrated an interaction between USP18 and YBX3 in these cells (Figure 5C). Given that USP18 functions as a DUB, we further examined the impact of USP18 on YBX3 ubiquitination. IP results indicated that USP18 depletion significantly increased the total YBX3 ubiquitination level, while USP18 overexpression decreased YBX3 ubiquitination in 786-O and A498 (Figures 5D and 5E).

To assess whether USP18’s catalytic activity is necessary for removing ubiquitin from YBX3, we generated a human *USP18* C64A mutant and examined its effect on YBX3 ubiquitination in 786-O cells. IP analysis revealed that overexpressing *USP18* C64A failed to decrease YBX3 ubiquitination, indicating a loss of deubiquitinase function (Figure 5F). Furthermore, we investigated the effect of USP18 on YBX3 stability in these cells. Our findings revealed that USP18 knockdown promoted YBX3 degradation, whereas excessive USP18 expression extended the half-life of YBX3 (Figures 5G and 5H), as evidenced by western blotting. These results collectively suggest that USP18 stabilizes YBX3 by reducing its ubiquitination level.

### Overexpression of YBX3 restores the mitigated malignant ability of USP18-deficient ccRCC cells

**Figure 6:**
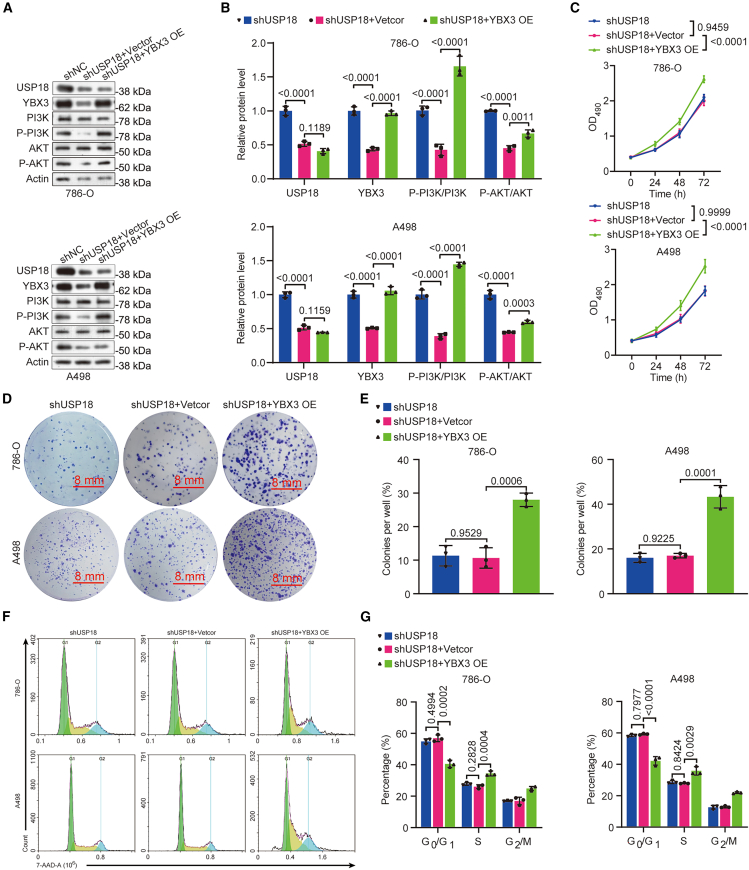
YBX3 overexpression increases P-PI3K and P-AKT levels and improves the viability, colony formation ability, and cell cycle progression in USP18-deficient ccRCC cells (A and B) Western blotting data demonstrate that YBX3 overexpression increases P-PI3K and P-AKT levels in USP18-deficient 786-O and A498. (C) MTT results show that excessive YBX3 enhances the viability of ccRCC cells with USP18 deficiency. (D and E) Colony formation assay outcomes suggest that YBX3 overexpression improves the proliferation of USP18-deficient ccRCC cells (scale bars, 8 mm). (F and G) Results from the 7-AAD staining assay demonstrate that YBX3 overexpression facilitates cell cycle progression in USP18-deficient ccRCC cells. One-way ANOVA followed by Tukey’s multiple comparison test was utilized for *p*-value calculation. The experiments were repeated three times independently. Error bars in Figures 6B, 6C, 6E, and 6G indicate standard deviation. Data are represented as mean ± SD.

To ascertain whether the carcinogenic effects of USP18 in ccRCC cells are mediated by YBX3, we conducted rescue experiments by overexpressing YBX3 in USP18-deficient ccRCC cells. Western blotting confirmed that the reduced YBX3 protein level induced by USP18 depletion could be restored by YBX3 overexpression (Figures 6A and 6B). MTT assay demonstrated that excessive YBX3 significantly enhanced the viability of USP18-deficient ccRCC cells (Figure 6C). Consistently, YBX3 overexpression improved the proliferation of ccRCC cells with USP18 deficiency, as indicated by the colony formation assay (Figures 6D and 6E).

**Figure 7:**
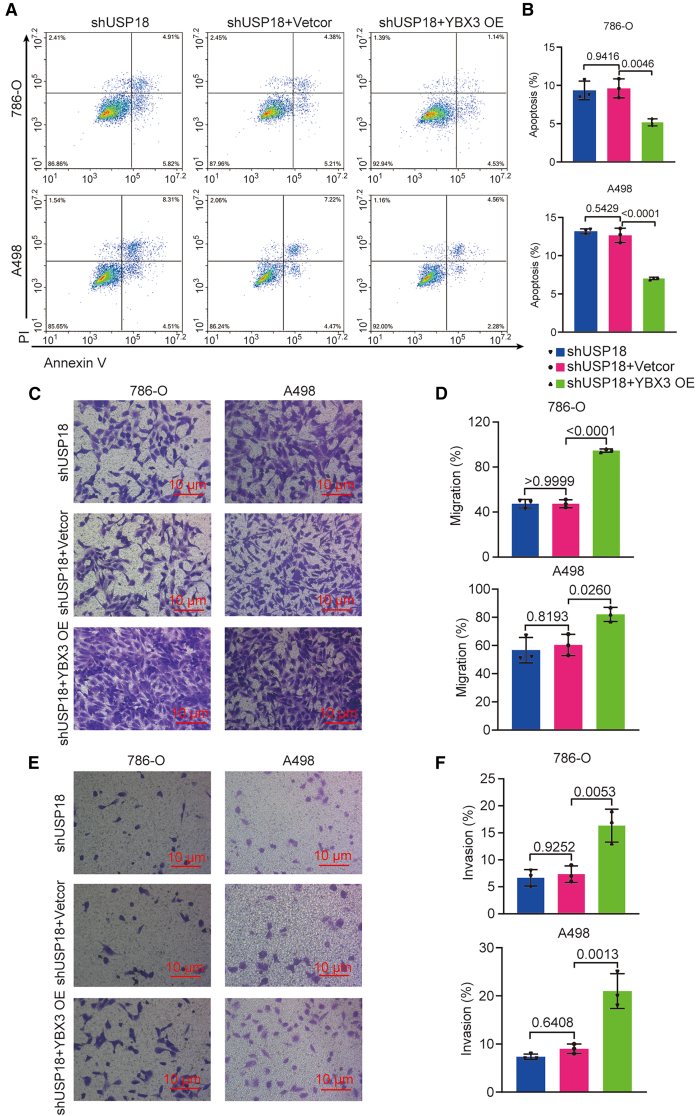
Overexpressing YBX3 alleviates apoptosis and promotes the migration and invasion of ccRCCs with USP18 deficiency (A and B) Annexin V/PI staining results show that the overexpression of YBX3 reduces the apoptosis of USP18-deficient ccRCC cells. (C–F) Transwell data uncover that excessive YBX3 stimulates the migration (C, D) and invasion (E, F) of USP18-deficient 786-O and A498 cells (scale bars, 10 μm). One-way ANOVA followed by Tukey’s multiple comparison test was used. The experiments were repeated three times independently. Error bars in Figures 7B, 7D, and 7F indicate standard deviation. Data are represented as mean ± SD.

Furthermore, 7-AAD staining revealed that cell-cycle arrest caused by USP18 knockdown was attenuated by YBX3 overexpression (Figures 6F and 6G). Additionally, Annexin V/PI staining and Transwell results respectively showed that excessive YBX3 reduced apoptosis and enhanced the migration and invasion of USP18-deficient ccRCC cells (Figures 7A–7F). At the molecular level, overexpression of YBX3 markedly elevated the levels of P-PI3K and P-AKT in USP18-deficient ccRCC cells, as illustrated by western blotting (Figures 6A and 6B). Collectively, these findings provide compelling evidence that YBX3 is crucial for USP18 to fulfill its oncogenic function in ccRCC cells.

### Knockdown of USP18 restrains ccRCC growth *in vivo*

**Figure 8:**
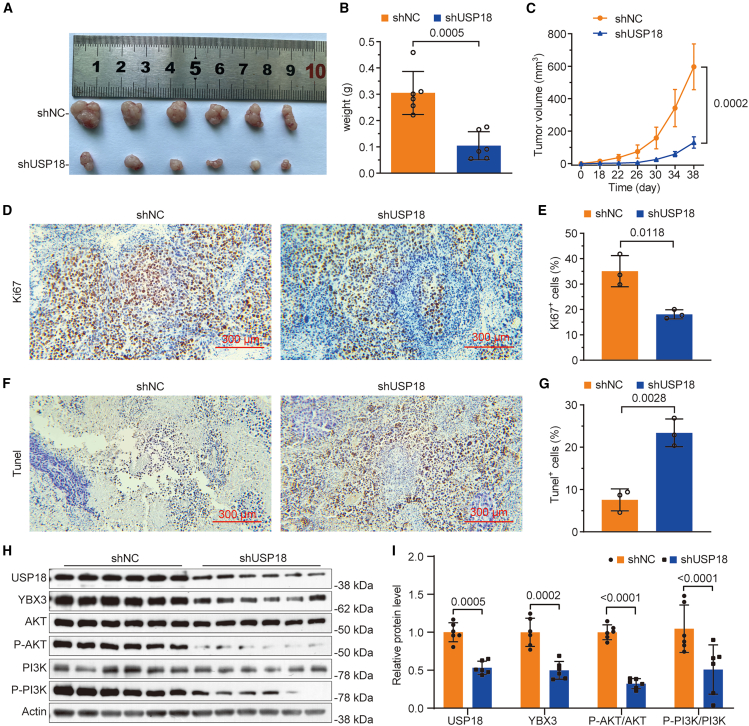
Knockdown of USP18 inhibits the progression of ccRCC *in vivo* (A) Whole-mount view of xenograft tumors developed from control and USP18-deficient 786-O cells (the ruler represents mm). (B and C) Curves depict the weight and volume of control and USP18-deficient xenograft tumors. (D and E) IHC data demonstrate that control tumor cells exhibit higher proliferation rates than USP18-deficient tumor cells (scale bars, 300 μm). (F and G) Results from the TUNEL assay reveal that USP18-deficient tumor cells display higher apoptotic rates than control tumor cells (scale bars, 300 μm). (H and I) Western blot data illustrate lower levels of USP18, YBX3, P-AKT, and P-PI3K in tumors developed from USP18-deficient 786-O cells compared to those from control. Band intensities were analyzed using ImageJ. The experiment was divided into two groups, including the shNC and shUSP18 groups, with 6 nude mice in each group. Unpaired two-tailed Student’s *t* test was employed for *p*-value calculation. Error bars in Figures 8B, 8E, 8G, and 8I indicate standard deviation. Error bars in Figure 8C indicate the standard error of the mean. Data are represented as mean ± SD, *n* = 6/group.

To assess the role of USP18 in ccRCC progression *in vivo*, a xenograft mouse model was constructed using control or *USP18*-deficient stable 786-O cells. The measurement of xenograft tumor weight and volume revealed a significant repression of tumor growth in mice implanted with USP18-deficient cells (Figures 8A–8C). This observation was further supported by the IHC analysis of Ki-67, a proliferation marker, which showed reduced proliferation in tumors derived from USP18-deficient 786-O cells compared to control (Figures 8D and 8E). Moreover, TUNEL results demonstrated an increased number of apoptotic cells in tumors developed from USP18-deficient 786-O cells compared with control (Figures 8F and 8G). At the molecular level, western blotting confirmed reduced levels of USP18, YBX3, P-PI3K, and P-AKT in tumors derived from USP18-deficient cells (Figures 8H and 8I). These findings illustrate that USP18 promotes ccRCC progression *in vivo*, likely through the stabilization of YBX3.

### USP18 deficiency inhibits the growth of ccRCC organoids

**Figure 9:**
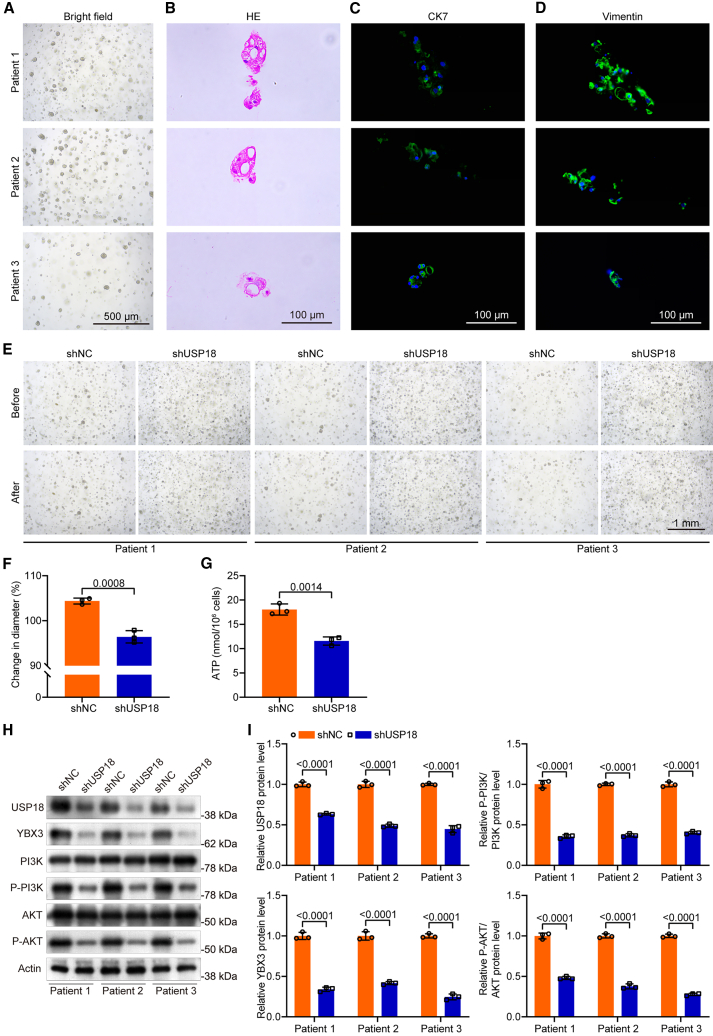
USP18 deficiency inhibits the growth of ccRCC organoids (A and B) Bright-field (A, scale bars, 500 μm) and H&E (B, scale bars, 100 μm) images showing the morphology and structure of ccRCC organoids from three patient samples. (C and D) IF data show the expression of CK7 (C) and Vimentin (D) in ccRCC organoids (scale bars, 100 μm). (E) Bright-field images showing the morphology of control and USP18-deficient ccRCC organoids. “Before” indicates the bright-field image of the organoids after transfection with shNC/shUSP18 at 72 h, and “after” indicates the bright-field image of the organoids after transfection with shNC/shUSP18 at 96 h. The images taken before and after are of the same organoids at different time points in the same field of view. (F) Change in diameter (%) of control and USP18-deficient ccRCC organoids. (G) ATP assay indicating the viability of control and USP18-deficient ccRCC organoids. (H and I) Western blot data indicate the expression of USP18, YBX3, P-AKT, and P-PI3K in control and USP18-deficient ccRCC organoids. Unpaired two-tailed Student’s *t* test was employed for *p*-value calculation. Data are represented as mean ± SD.

To further investigate the role of USP18 in human ccRCC, we utilized ccRCC organoids derived from primary ccRCC tissues. The organoids exhibited typical organoid morphology and structure (Figures 9A and 9B), expressing CK7 and Vimentin (Figures 9C and 9D). The size change of USP18-deficient organoids was visibly less pronounced compared to controls (Figures 9E and 9F). ATP quantification assays showed significantly reduced viability and proliferation in USP18-deficient organoids compared to controls (Figure 9G). Western blot analysis confirmed that YBX3 expression was significantly reduced in USP18-deficient ccRCC organoids, with decreased levels of P-PI3K and P-AKT expression (Figures 9H and 9I). These data further confirm the oncogenic role of USP18 in ccRCC.

## Discussion

While significant progress has been made in understanding the pathology of ccRCC,^27^^,^^28^ the mechanism of development and progression of this fatal disease remains incompletely understood. The critical role of ubiquitination regulators in ccRCC has garnered recognition.^29^^,^^30^ Consistently, our data showing USP18 overexpression in ccRCC and its oncogenic function support the notion that targeting the ubiquitin-proteasome system may offer benefits for ccRCC treatment.

USP18 is well known for suppressing type I interferon signaling by acting as a scaffold: It binds STAT2 and helps recruit it to the IFNAR2 receptor subunit, thereby blocking downstream JAK1 activation.^31^ In addition to this scaffold function, USP18 has enzymatic activity that removes the ubiquitin-like modifier ISG15 from ISG15-conjugated (ISGylated) proteins. Mutations that abolish this catalytic function, such as the C64S or C64A substitutions in human *USP18*, greatly impair its capacity to mediate deISGylation.^32^

Beyond regulating immune response, USP18’s role in various cancers has also been extensively documented. For example, it facilitates the growth of breast cancer by stimulating the EGFR/AKT/Skp2 pathway,^26^ exerts carcinogenic effects in cervical cancer through activating the AKT signaling cascade,^25^ and promotes lung cancer growth, possibly via stimulating lipolysis and fatty acid oxidation.^33^ While the oncogenic role of USP18 has often been linked to its immunomodulatory activity,^32^ increasing evidence indicates that its overlooked deubiquitinase function also contributes to malignancy. For instance, USP18 can bolster colon cancer and neck squamous cell carcinoma progression by deubiquinating and stabilizing Snail1 and PLK1, respectively.^34^^,^^35^ These findings align with our discovery that USP18 acts as an oncogene in ccRCC by upregulating YBX3 protein level through the ubiquination pathway. Importantly, the *USP18* C64A mutant is unable to remove ubiquitin from YBX3, demonstrating that the catalytic domain is essential for both its deISGylation and deubiquitination activities. It would be intriguing to determine whether USP18 promotes tumorigenesis in other cancers by stabilizing YBX3, especially considering YBX3’s oncogenic role in various cancers, including colon and breast cancer.^23^^,^^36^ Conversely, whether USP18 regulates other substrates in ccRCC also warrants further investigation.

The carcinogenic effects of USP18 may depend on its ability to stabilize cyclin D1,^37^ which promotes cancer cell proliferation.^38^ Moreover, USP18 may promote tumorigenesis by activating the PI3K/AKT pathway, a well-characterized oncogenic signaling.^16^ Our finding that *USP18* deficiency leads to decreased PI3K/AKT activity suggests that this pathway may be crucial for USP18’s oncogenic role in ccRCC. While the mechanism by which USP18 activates the PI3K/AKT pathway remains elusive, our results highlight YBX3 as its overexpression restores the reduced level of PI3K/AKT signaling in USP18-deficient ccRCC cells. Further exploration is warranted to uncover the precise mechanism.

USP18 is frequently overexpressed in multiple malignancies, including colon cancer and head-and-neck squamous cell carcinoma.^34^^,^^35^ In line with these reports, our findings demonstrate that both *USP18* mRNA and protein levels are substantially increased in human ccRCC samples. However, the underlying mechanisms driving this upregulation remain uncertain. Altered promoter methylation has been linked to aberrant *USP18* expression in other scenarios,^26^^,^^39^ and interferon signaling is also known to induce *USP18* transcription.^40^ Whether either of these processes contributes to its elevated expression in ccRCC requires further characterization.

YBX3 is an RNA-binding protein that regulates the expression of its target genes at the posttranslational level.^41^^,^^42^ On the other hand, the mechanism underlying YBX3 expression regulation remains poorly understood. Given its frequent overexpression in cancer cells and its correlation with poor prognosis, it is imperative to understand the regulatory mechanisms governing YBX3 expression. Our data demonstrating the interaction between USP18 and YBX3 in ccRCC cells, and the subsequent downregulation of YBX3 ubiquitination by USP18, suggest that dysregulated ubiquitination regulators contribute to YBX3 overexpression in cancer cells. This dysregulation may result from increased expression of YBX3 stabilizers such as USP18 or decreased expression of USPs that facilitate YBX3 degradation. Indeed, our screening data identified multiple USPs that negatively influence YBX3 stability. However, this result requires further validation and functional analysis.

Despite USP18’s role in reducing the ubiquitination of YBX3, the specific types of ubiquitination (e.g., K48, K63, or other non-canonical types) regulated by USP18 remain unclear. Moreover, the domain of USP18 that interacts with YBX3, and the residues on YBX3 mediating its interaction with USP18, have not been explored. This gap is noteworthy because USP18 can modulate substrate ubiquitination by serving as a scaffold that recruits other DUBs. For example, USP18 facilitates the innate antiviral response by bringing USP20 to STING/MITA, enabling its deubiquitination.^43^ While our preliminary luciferase-based screen for YBX3 regulators showed that USP20 overexpression actually reduced YBX3 levels, it remains possible that USP18 collaborates with additional USPs to control YBX3 ubiquitination and stability. This idea warrants further investigation. Additionally, the enzymes responsible for ubiquitinating YBX3 have yet to be identified. Finally, it will be important to clarify whether USP18’s oncogenic function in ccRCC is linked to its modulation of interferon signaling and ISG15.

In conclusion, this research unveils the carcinogenic effects of the USP18-YBX3 axis in ccRCC. These findings offer fresh insights into the involvement of ubiquitination regulators in ccRCC pathogenesis and may lay the groundwork for the development of novel targeted therapies against this lethal disease.

### Limitations of the study

This study has several limitations. Although the current research results are based on *in vivo* animal models as well as *in vitro* cell and organoid models, further validation in clinical human samples is needed to enhance the translational potential of the USP18-YBX3 axis in clinical diagnosis and treatment. In addition, the underlying mechanisms driving the upregulation of USP18 in ccRCC remain to be further elucidated. The specific type of ubiquitination regulated by USP18, the domains through which USP18 interacts with YBX3, and the residues on YBX3 that mediate its interaction with USP18 also require further investigation to strengthen the robustness of the research findings.

## Resource availability

### Lead contact

Further information and requests for resources should be directed to and will be fulfilled by the correspondence contact, Chen Wang (E-mail: l-morning@fjmu.edu.cn).

### Materials availability

This study did not generate new unique reagents.

### Data and code availability

• Data: Data reported in this paper will be shared by the lead contact upon request.
• Code: This paper does not report original code.
• Additional Information: Any additional information required to reanalyze the data reported in this paper are available from the lead contact upon request. Publicly available datasets used in this study were accessed from the Gene Expression Omnibus (GEO) database (https://www.ncbi.nlm.nih.gov/gds). Accession numbers are listed in the key resources table.

## Acknowledgments

We are grateful for the funding support from the General Program of Fujian Natural Science Foundation (no. 2023J011158).

## Author contributions

CW, YHH and ZJY conducted all the experiments in this study, SQC and X.C. wrote the overall article and plotted the data, CW conceived and designed this study, as well as revised the manuscript, YHH, ZJY and X.C. (the last author) conducted statistical analysis of all the results of the article. All the authors reviewed the final manuscript and agreed to submit it for publication.

## Declaration of interests

The authors declare that they have no conflicts of interest.

## STAR★Methods

### Key resources table

**Table:** 

REAGENT or RESOURCE	SOURCE	IDENTIFIER
**Antibodies**		

USP18 Rabbit Polyclonal antibody	Proteintech	12153-1-AP; RRID:AB_2877830
CSDA (YBX3) Rabbit Polyclonal antibody	Proteintech	27785-1-AP; RRID:AB_3085996
AKT Mouse Monoclonal antibody	Proteintech	60203-2-Ig; RRID:AB_10912803
Phospho-AKT (Ser473) Recombinant Rabbit monoclonal antibody	Proteintech	80455-1-RR; RRID:AB_2918892
PI3 Kinase p110 Beta Rabbit Polyclonal antibody	Proteintech	20584-1-AP; RRID:AB_10734439
DYKDDDDK tag Rabbit Polyclonal antibody	Proteintech	20543-1-AP; RRID:AB_11232216
Beta Actin Rabbit Polyclonal antibody	Proteintech	20536-1-AP; RRID:AB_10700003
HRP-conjugated Goat Anti-Rabbit IgG(H+L)	Proteintech	SA00001-2; RRID:AB_2722564
HRP-conjugated Goat Anti-Mouse IgG(H+L)	Proteintech	SA00001-1; RRID:AB_2722565
Phospho-PI3 Kinase p85 (Tyr458)/p55 (Tyr199) (E3U1H) Rabbit Monoclonal Antibody	Cell Signaling Technology	#17366; RRID:AB_2895293
Ki-67 (8D5) Mouse Monoclonal Antibody	Cell Signaling Technology	#9449; RRID:AB_2797703
Cytokeratin 7 Polyclonal antibody	Proteintech	15539-1-AP; RRID:AB_2249769
Vimentin Polyclonal antibody	Proteintech	10366-1-AP; RRID:AB_2273020

**Bacterial and viral strains**		

pmirGLO-G4S-Nanoluc	Anti-Hela Biotechnology	N/A
pLKO.1-puro	Anti-Hela Biotechnology	N/A

**Biological samples**		

Human ccRCC tissue	This paper	N/A
Human adjacent non-cancerous tissue	This paper	N/A

**Chemicals, peptides, and recombinant proteins**		

Y27632	Immocell	IMC-014-Y
MG132	MedChemExpress	HY-13259
Cycloheximide (CHX)	MedChemExpress	HY-12320
Fetal bovine serum	Gibco	A5256701
Lipofectamine 2000	Invitrogen	11668027
DNase-free proteinase K	Beyotime	ST532
Puromycin	Yeasen	60209ES50
BeyoMag™ Protein A+G beads	Beyotime	P2108

**Critical commercial assays**		

FastPure Cell/Tissue Total RNA Isolation Kit V2	Vazyme	RC112
HiScript II Q RT SuperMix for qPCR	Vazyme	R222-01
ChamQ SYBR Color qPCR Master Mix (Low ROX Premixed)	Vazyme	Q431-02
Dual Luciferase Reporter Assay Kit	Vazyme	DL101
Pierce™ BCA Protein Assay Kits	Thermo Fisher Scientific	#23227
MTT solution	Yeasen	#40201ES72
Annexin V-FITC/PI apoptosis detection kit	Vazyme	A211-01
TUNEL detection kit	Beyotime	C1091
ATP detection kit	Beyotime	S0027

**Deposited data**		

Raw WB data	This paper	N/A
GSE40435	NCBI GEO	https://www.ncbi.nlm.nih.gov/geo/query/acc.cgi?acc=GSE40435
GSE126964	NCBI GEO	https://www.ncbi.nlm.nih.gov/geo/query/acc.cgi?acc= GSE126964

**Experimental models: Cell lines**		

Caki-1 cell	Immocell	IM-H364
A498 cell	Immocell	IM-H056
Caki-2 cell	Immocell	IM-H266
786-O cell	Immocell	IM-H055
HK-2 cell	Immocell	IM-H060
HEK293T cell	Immocell	IM-H222

**Experimental models: Organisms/strains**		

Female BALB/c nude mice	This paper	N/A

**Oligonucleotides**		

Human *USP18*-Forward	This paper	5’-TGGACAGACCTGCTGCCTTAAC-3’
Human *USP18*-Reverse	This paper	5’-CTGTCCTGCATCTTCTCCAGCA-3’
Human *YBX3*-Forward	This paper	5’-TGGTCCAAACCAGCCGTCTGTT-3’
Human *YBX3*-Reverse	This paper	5’-GTTCTCAGTTGGTGCTTCACCTG-3’
Human *18s rRNA*-Forward	This paper	5’-ACCCGTTGAACCCCATTCGTGA-3’
Human *18s rRNA*-Reverse	This paper	5’-GCCTCACTAAACCATCCAATCGG-3’
HA-USP18-Forward	This paper	5’-TAGAGAATTCGGATCCATGAGCAAG GCGTTTGGGCTC-3’
HA-USP18-Reverse	This paper	5’-AGCTTCCATGGCTCGAGTTAGCAC TCCATCTTCATG-3’
Flag-YBX3-Forward	This paper	5’-TACCGAGCTCGGATCCGCCACCAT GAGTGAGGCGGGCGAG-3’
Flag-YBX3-Reverse	This paper	5’-GCCCTCTAGACTCGAGCTCAGCAC TGCTCTGCTGGG-3’
shUSP18-1-Forward	This paper	5’-CCGGAGAAGGAAGAAGACAGCAACACT CGAGTGTTGCTGTCTTCTTCCTTCTTTTTT-3’
shUSP18-1-Reverse	This paper	5’-AATTAAAAAAGAAGGAAGAAGACAGCAAC ACTCGAGTGTTGCTGTCTTCTTCCTTCT-3’
shUSP18-2-Forward	This paper	5’-CCGGGGAATTCACAGACGAGAAAGACTC GAGTCTTTCTCGTCTGTGAATTCCTTTTT-3’
shUSP18-2-Reverse	This paper	5’-AATTAAAAAGGAATTCACAGACGAGAAAG ACTCGAGTCTTTCTCGTCTGTGAATTCC-3’
shUSP18-3-Forward	This paper	5’-CCGGGGTCATTACTGTGTCTACATCCTCG AGGATGTAGACACAGTAATGACCTTTTT-3’
shUSP18-3-Reverse	This paper	5’-AATTAAAAAGGTCATTACTGTGTCTACATCC TCGAGGATGTAGACACAGTAATGACC-3’
shNC-Forward	This paper	5’-CCGGTTCTCCGAACGTGTCACGTTTCTCGA GAAACGTGACACGTTCGGAGAATTTTT-3’
shNC-Reverse	This paper	5’-AATTAAAAATTCTCCGAACGTGTCACGTTTC TCGAGAAACGTGACACGTTCGGAGAA-3’
HA-USP18 C64A-Forward	This paper	5’-GGACAGACCGCATGCCTTAACTCCTTGATTCAGG-3’
HA-USP18 C64A-Reverse	This paper	5’-TTAAGGCATGCGGTCTGTCCAATGTTGTGTAAACC-3’

**Software and algorithms**		

GraphPad Prism	GraphPad	Version 8.0
R software	CRAN	Version 4.3.0
ImageJ	NIH	Version 1.54
SPSS	IBM	Version 22.0

**Other**		

NovoCyte^TM^ flow cytometer	Agilent	N/A
CFX Connect 96 device	Bio-Rad	N/A
LMS 900 confocal microscope	Zeiss	N/A
GloMax® Navigator Microplate Luminometer	Promega	N/A
Inverted bright-field microscope	Motic	N/A
Microplate Luminometer Orion II	Berthold Technologies	N/A

### Experimental model and study participant details

#### Xenograft assay

The BALB/c nude mice (aged 4∼6 weeks, female) were acquired from Beijing Vital River Technology. Mice were reared in a specific pathogen-free environment with individual ventilated cages, alternating light and dark for 12 h, and allowed to eat freely. After adaptive housing, mice were stochastically divided into two groups, 6 mice in each group. 2 × 10^6^ control or USP18-deficient stable 786-O cells were injected into the right flank of each mouse. The length and width of the xenograft tumors were measured at the indicated time points. Volumes of tumors were calculated according to the formula: (length×width^2^)/2. 38 days after the initial injection, mice were euthanized, and the tumors were dissected, weighed, and photographed. Half of the tumors were fixed with 4% PFA for histology, and the remaining tumors were subjected to Western blotting.

#### Human tissues and cell lines

ccRCC samples containing tumor (T) and adjacent non-cancerous (N) tissue were collected from patients diagnosed and treated with ccRCC in Fujian Provincial Hospital. Human ccRCC cell lines Caki-1 (RRID: CVCL_0234), A498 (RRID: CVCL_1056), Caki-2 (RRID: CVCL_0235), 786-O (RRID: CVCL_1051), along with human immortalized proximal tubule epithelial cell line (HK-2) (RRID: CVCL_0302), immortalized human embryonic kidney cell line (HEK293T) (RRID: CVCL_0063), were acquired from the Immocell. All cells were cultured with their respective recommended media, along with 10% fetal bovine serum (FBS, Thermo Fisher Scientific, USA) and 5% CO_2_. Regular examinations were conducted to exclude mycoplasma contamination.

#### Ethics approval and consent to participate

This study was approved by the Ethics Committee of Fujian Provincial Hospital (No. K2024-06-036) and performed with written consent from patients, adhering to the principles of the Declaration of Helsinki. All animal experiments were approved by the Ethics Committee of Fujian Provincial Hospital (No. IACUC-FPH-SL-20230724 [0070]). All procedures were performed in accordance with the ARRIVE guidelines.

### Method details

#### Bioinformatics

Two transcriptomic datasets of ccRCC, GSE40435 and GSE126964, were accessed from the Gene Expression Omnibus (GEO) database (https://www.ncbi.nlm.nih.gov/gds). Differential expression of USP18 and YBX3 was assessed by the limma package (v3.56.2) in R (v4.3.0). For pathway enrichment analysis of differentially expressed genes associated with USP18 levels, the GSE126964 dataset was analyzed using the Pathway Interaction Database (PID) function of the R package “GSEA” (v4.2.1) based on USP18 expression. The top 30 pathways with FDR values less than 0.05 were visualized using the R package “ggplot2” (v3.5.0).

#### Generation of ccRCC organoids

Three human ccRCC specimens (approximately 0.25–1 cm^3^) were collected, and the tissues were washed with cold PBS containing penicillin/streptomycin. Then, the samples were finely cut into fragments approximately 1 mm × 1 mm in size. The minced tissues were placed in pre-warmed digestion solution and gently shaken repeatedly until most of the fragments were decomposed. The suspension was filtered through a 70 μm strainer to remove any remaining undigested tissue. The resulting cell clusters measured between 5–15 μm in diameter. Subsequently, the cell cluster suspension was centrifuged, and the pellet was resuspended in liquid Matrigel. Approximately 30–40 μL of the mixture was seeded into each well of 24-well plates, and the plate was incubated at 37 °C for 15–20 min to allow the Matrigel to solidify. Following this, 500 μL of ccRCC organoid culture medium supplemented with 10 μM Y27632 was added to each well. The organoids were maintained in a 37 °C, 5% CO_2_ incubator, with the culture medium replaced every two days.

#### Plasmid construction and lentivirus production

The forty USP overexpression plasmids were acquired from Anti-Hela Biotechnology (Xiamen, China). To construct the *YBX3*-Nanoluc vector, the coding sequence of *YBX3* was cloned into the pmirGLO-G4S-Nanoluc vector. For the knockdown of *USP18*, *USP18*-targeting shRNAs were inserted into the pLKO.1-puro lentiviral vector, resulting in plasmids named shUSP18-1, shUSP18-2, and shUSP18-3. Non-specific shRNA (shNC) was used for control. The His-Ub plasmid was obtained from Miaoling Technology (China). All plasmids underwent Sanger sequencing to confirm the accuracy of the DNA sequence. Non-viral cell transfection was performed using Lipofectamine 2000 (Invitrogen, USA). Lentivirus production was completed by Anti-Hela Biotechnology. The virus multiplicity of infection (MOI) was determined based on TaqMan real-time PCR, with the optimized MOI set at 20. Infected cells were cultured with 2 μg/mL puromycin for 7 d, with the medium replenished every other day to construct the stable cells. Finally, the surviving cells were maintained in puromycin-free medium for subsequent analysis. The primer sequences for plasmid construction are shown in Table 1.

**Table 1:** The primer sequences for plasmid construction

Target	Sequence (5′-3′)
HA-USP18-Forward	TAGAGAATTCGGATCCATGAGCAAGGCGTTTGGGCTC
HA-USP18-Reverse	AGCTTCCATGGCTCGAGTTAGCACTCCATCTTCATG
Flag-YBX3- Forward	TACCGAGCTCGGATCCGCCACCATGAGTGAGGCGGGCGAG
Flag-YBX3- Reverse	GCCCTCTAGACTCGAGCTCAGCACTGCTCTGCTGGG
shUSP18-1-Forward	CCGGAGAAGGAAGAAGACAGCAACACTCGAGTGTTGCTGTCTTCTTCCTTCTTTTTT
shUSP18-1-Reverse	AATTAAAAAAGAAGGAAGAAGACAGCAACACTCGAGTGTTGCTGTCTTCTTCCTTCT
shUSP18-2-Forward	CCGGGGAATTCACAGACGAGAAAGACTCGAGTCTTTCTCGTCTGTGAATTCCTTTTT
shUSP18-2-Reverse	AATTAAAAAGGAATTCACAGACGAGAAAGACTCGAGTCTTTCTCGTCTGTGAATTCC
shUSP18-3-Forward	CCGGGGTCATTACTGTGTCTACATCCTCGAGGATGTAGACACAGTAATGACCTTTTT
shUSP18-3-Reverse	AATTAAAAAGGTCATTACTGTGTCTACATCCTCGAGGATGTAGACACAGTAATGACC
shNC-Forward	CCGGTTCTCCGAACGTGTCACGTTTCTCGAGAAACGTGACACGTTCGGAGAATTTTT
shNC-Reverse	AATTAAAAATTCTCCGAACGTGTCACGTTTCTCGAGAAACGTGACACGTTCGGAGAA
HA-USP18 C64A-Forward	GGACAGACCGCATGCCTTAACTCCTTGATTCAGG
HA-USP18 C64A-Reverse	TTAAGGCATGCGGTCTGTCCAATGTTGTGTAAACC

For lentiviral infection of ccRCC organoids, the organoids were collected and resuspended in 500 μL of complete organoid medium containing 50 μL of a 1: 50 diluted lentiviral suspension (titer=1 × 10^7^) and mixed thoroughly. The mixture was transferred to a well of a 24-well low-attachment plate and incubated for 24 h. Then organoid medium supplemented with 1 μg/mL puromycin was added for selection. The surviving organoids were collected for further analysis.

#### Dual-luciferase assay

To measure luciferase activity, we used the Dual Luciferase Reporter Assay Kit (#DL101, Vazyme, China). HEK293T cells were transfected with various USP overexpression plasmids utilizing Lipofectamine 2000 (Invitrogen). After a 48-hour incubation period, the cells were washed and lysed with the kit's lysis buffer for 5 min at room temperature (RT). Then lysates were collected and centrifuged, and 20 μL of supernatant was combined with 100 μL of Firefly substrate solution. Firefly luciferase activity was detected by a GloMax® Navigator Microplate Luminometer (Promega, USA) immediately after mixing. Next, 100 μL of Renilla substrate solution was added to the same mixture, and luciferase activity was detected by the luminometer (Promega) immediately. The luminescence detector collects all light in the entire visible spectrum. Firefly luciferase activity was normalized to the corresponding Renilla luciferase activity.

#### Western blotting

Total protein was prepared using RIPA lysis buffer containing protease inhibitors. Protein concentration was quantified by Pierce™ BCA kit (#23227, Thermo Fisher Scientific), and absorbance values were determined at a wavelength of 562 nm. Subsequently, 15 μg of each sample was separated by 10% SDS-PAGE and transferred onto PVDF membranes. Next, membranes were blocked with 5% non-fat milk/TBS for 1 h and then probed with primary antibody at 4°C. Then membranes were washed and incubated with secondary antibody solutions for 1 h at RT. Finally, membranes were washed and incubated with enhanced chemiluminescence (ECL) reagents (Millipore, USA) to visualize the signal using by Bio-Rad detection system. ImageJ (NIH, USA) was employed to analyze the band intensity. The antibodies used are shown in Table 2.

**Table 2:** Antibodies for Western blotting

Antibody	Resource	Cat. No.	Dilution
USP18	Proteintech	12153-1-AP	1:1000
YBX3	Proteintech	27785-1-AP	1:1000
AKT	Proteintech	60203-2-Ig	1:3000
P-AKT	Proteintech	80455-1-RR	1:3000
PI3K	Proteintech	20584-1-AP	1:1000
Flag	Proteintech	20543-1-AP	1:3000
Actin	Proteintech	20536-1-AP	1:5000
HRP-Anti-Rabbit IgG	Proteintech	SA00001-2	1:10000
HRP-Anti-Mouse IgG	Proteintech	SA00001-1	1:10000
P-PI3K	CST	#17366	1:500

#### Quantitative PCR (qPCR)

Cells of 80% confluence were harvested for total RNA isolation by FastPure cell/tissue total RNA isolation kit V2 (#RC112, Vazyme). The HiScript II Q RT SuperMix for qPCR (R222-01, Vazyme) was employed for reverse transcription. qPCR was performed by ChamQ SYBR Color qPCR Master Mix (Low ROX Premixed) (Q431-02, Vazyme) on a CFX Connect 96 device (Bio-Rad, USA). The 2^−ΔΔCt^ method was utilized to determine the relative expression of target genes. qPCR primers are listed in Table 3

**Table 3:** The qPCR sequence

Target	Sequence (5′-3′)
*USP18*-Forward	TGGACAGACCTGCTGCCTTAAC
*USP18*-Reverse	CTGTCCTGCATCTTCTCCAGCA
*YBX3*-Forward	TGGTCCAAACCAGCCGTCTGTT
*YBX3*-Reverse	GTTCTCAGTTGGTGCTTCACCTG
*18s rRNA*-Forward	ACCCGTTGAACCCCATTCGTGA
*18s rRNA*-Reverse	GCCTCACTAAACCATCCAATCGG

.

#### Colony formation assay

Cells were seeded into 6-well plates (200/per well), and cultured for 14 d, with the medium being refreshed every other day. After that, the medium was removed and the cell was rinsed with PBS, followed by fixation with 4% paraformaldehyde (PFA) for 20 min. Subsequently, the PFA was discarded, and cells were rinsed with PBS again and stained with 0.1% crystal violet for 5 min at RT. Finally, the staining solution was removed, cells were rinsed with PBS, and stained cell colonies were photographed and then quantified using ImageJ.

#### MTT assay

Cells were seeded into a 96-well plate (1×10^4^/per well). After 0, 24, 48, or 72 h, cells were incubated with 50 μL MTT solution (Yeasen, Shanghai, China) for another 4 h. Subsequently, the medium was removed, and 150 μL DMSO was added to dissolve the crystal. Finally, the solutions were subjected to measurement of OD_490_ values using a microplate reader.

#### Transwell assay

For migration assay, cells resuspended in serum-free medium were seeded into upper chambers of Transwell (8 μm pores, Corning, 1.5×10^5^ /per well), and the lower chambers were filled with medium containing 10% FBS. After 24 h, cells were fixed with 4% PFA, and the Transwell membranes were dissected, stained with 0.1% crystal violet, and photographed. The stained cell numbers were quantified using ImageJ. For invasion assay, the upper chambers were precoated with Matrigel (Becton Dickinson Biosciences). All other procedures were identical to the migration assay.

#### Cell cycle assay

Cells were harvested, resuspended in ice-cold PBS, and then fixed with 70% cold ethanol at 4°C overnight. Subsequently, the fixed cells were incubated with RNase A staining solution for 30 min in the dark. After removing the RNase A solution, cells were resuspended with PBS containing 5 μL 7-AAD solution (Thermo Fisher), and incubated in a light-protective environment at 4°C for 30 min. Next, the cells were stained with propidium iodide (PI) reagent for 30 min and then subjected to flow cytometry analysis using a NovoCyte^TM^ flow cytometer.

#### Annexin V/PI staining

Annexin V-FITC/PI apoptosis detection kit (#A211-01, Vazyme) was applied to detect cell apoptosis. Cells were initially resuspended at a density of 6×10^5^ cells/mL. Then cells were incubated with Annexin V-FITC and PI solution and subjected to flow cytometry analysis.

#### Co-immunoprecipitation (Co-IP)

2 μg anti-Flag or anti-HA antibody (Proteintech, China) was incubated with BeyoMag™ Protein A+G beads (Beyotime, China) for 10 min. Subsequently, the beads were washed and mixed with protein samples, gently shaking for 2 h at RT. Next, the beads were pelleted and washed, incubated with 20 μL elution buffer, and the resulting elution was collected for subsequent Western blotting.

#### Immunohistochemistry (IHC)

The xenograft tumor tissues were paraffin-embedded and sectioned into 5 μm slices. After deparaffinization, the slices were treated with 3% H_2_O_2_ for 15 min, followed by antigen retrieval. Next, the slices were blocked with 5% goat serum and incubated with PBST containing anti-Ki67 (#9449, Cell Signaling Technology, USA, 1:1000) at 4°C. The slices were washed and incubated with HRP-conjugated goat anti-mouse IgG (PN0080, Pinuofei, China, 1:500) for 1 h at RT. Finally, the slices were washed and subjected to DAB staining, counterstaining with Meyer’s hematoxylin, dehydration, and mounting. The IHC results were observed and photographed with a microscope. The percentage of positive cells of Ki-67 was quantified using ImageJ.

TUNEL assays were conducted using a TUNEL kit (C1091, Beyotime, China). In brief, paraffin sections were deparaffinized in xylene for 10 min, followed by rehydration using gradient ethanol. After washing with PBS, the sections were digested with DNase-free proteinase K (#ST532, Beyotime, 20 μg/mL) at 37°C for 20 min. Next, the sections were incubated with endogenous peroxidase blocking solution (#P0100B, Beyotime) for 20 min at RT and then treated with 50 μL biotinylated solution at 37°C for 60 min in the dark. Subsequently, the sections were sequentially incubated with 0.2 mL of reaction termination solution for 10 min and 50 μL streptavidin-HRP working solution for 30 min, followed by three washes of PBS. Afterward, the sections were incubated with 0.3 mL DAB solution for 20 min, followed by counterstaining with hematoxylin staining solution (#C0107, Beyotime). Finally, the sections were washed with water and dehydrated with gradient ethanol, cleared with xylene, mounted, and imaged. The data were quantified by ImageJ.

#### Hematoxylin and eosin (H&E) staining

Organoids were collected and made into paraffin sections. The sections were immersed in xylene and then sequentially rehydrated through graded ethanol solutions and double-distilled water. The slices were then stained in H&E staining solution 1 for 3 min, H&E staining solution 2 for 3 s, and H&E staining solution 3 for 3 s, followed by rapid water washing. Then slices were immersed in 85% ethanol, 95% ethanol, H&E staining solution 4, absolute ethanol I-III, n-butyl alcohol, and xylene I-II for 3 min, sequentially. Finally, the slices were air-dried and sealed with neutral gum.

#### Immunofluorescence (IF)

Organoids were collected and fixed in 4% paraformaldehyde before being processed into 5 μm frozen sections. The sections were washed with PBS and blocked with 1% BSA for 1 h. Then, they were incubated overnight with primary antibodies, including anti-human CK7 (15539-1-AP, Proteintech, 1: 500) or anti-human Vimentin (10366-1-AP, Proteintech, 1: 500). Afterwards, the sections were washed 3 times with PBST. They were then incubated in the dark for 1 h with fluorescent secondary antibody solution. Afterward, the sections were washed again with PBST, mounted using a DAPI-containing medium, and imaged using an LMS 900 confocal microscope (Zeiss, Germany).

#### Organoids size measurement

The morphology of organoids was examined using an inverted bright-field microscope (Motic, China) equipped with a digital camera. The diameters of these organoids were measured using the associated software.

#### ATP concentration measurement

The ATP levels were assessed by a kit (S0027, Beyotime), following the manufacturer’s instructions using a Microplate Luminometer Orion II (Berthold Technologies, Germany).

### Quantification and statistical analysis

Statistical analysis and plot generation were conducted using SPSS v22.0 and GraphPad Prism v8.0. The data are presented as mean ± standard deviation (SD). The methods for *p*-value calculation were detailed in the figure legends. *p* < 0.05 was considered statistically significant. All experiments were independently performed at least 3 times. Comparisons between two groups were performed using an unpaired two-tailed Student’s *t* test, and comparisons among multiple groups were analyzed by One-way ANOVA followed by Tukey’s post hoc test. For experiments involving two independent variables, Two-way ANOVA followed by Sidak's post hoc test was used.
