# Supplementary material for: USP18 promotes clear cell renal cell carcinoma progression by regulating the ubiquitination and stability of YBX3
Source: iScience. 2026 Apr 17;29(5):115808. doi: 10.1016/j.isci.2026.115808 (PMC13157185; doi:10.1016/j.isci.2026.115808)
Supplement: Document S1. Figures S1 and S2 [file mmc1.pdf]

**iScience, Volume *29***

## Supplemental information

# USP18 promotes clear cell renal cell carcinoma progression by regulating the ubiquitination and stability of YBX3

**Chen Wang, Yihui He, Zhijie You, Siqi Chen, Xin Chen, and Xin Chen**

## Supplementary Figures

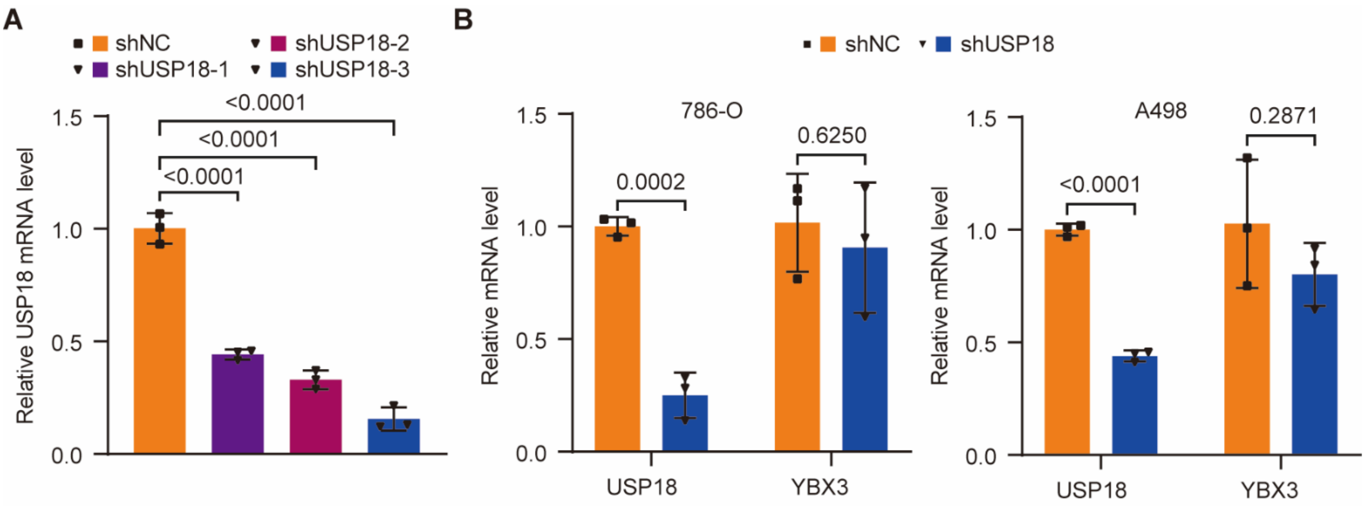


**Supplementary Figure 1.** Validation of USP18 knockdown efficiency. Data are represented as mean +/- SD.

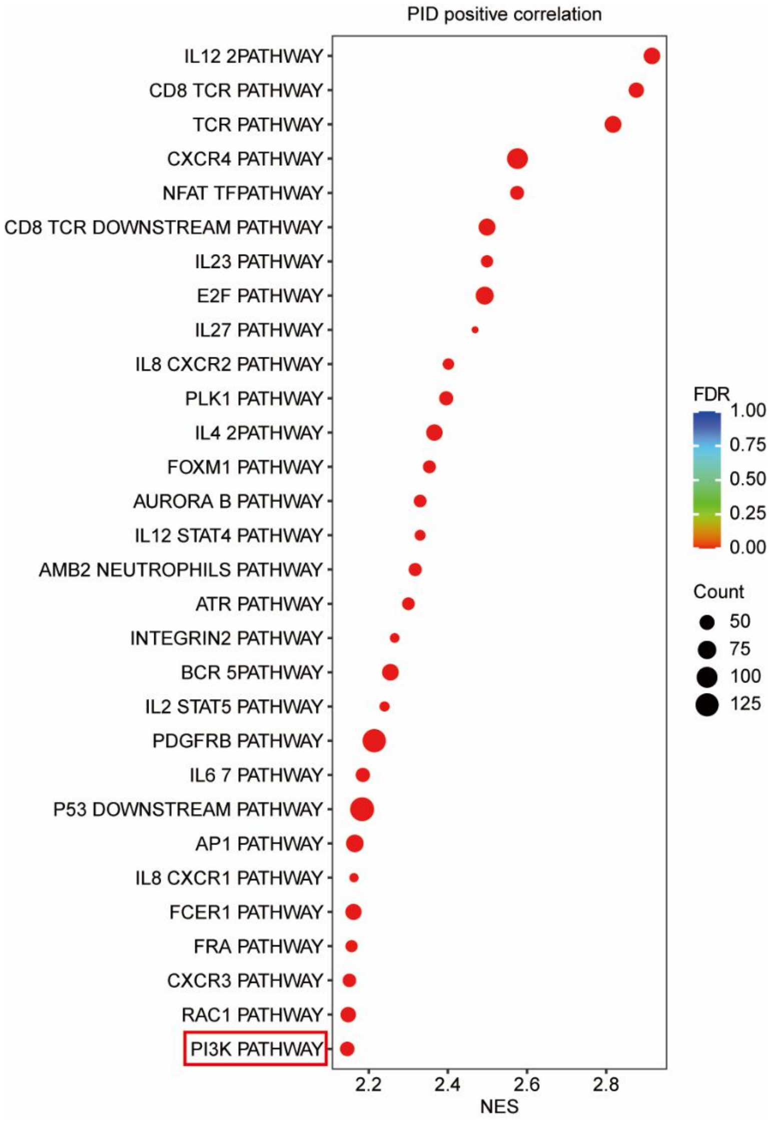


**Supplementary Figure 2.** Pathway enrichment analysis of the differentially expressed genes associated with *USP18* expression in ccRCC.
